# Bond Strength of 10-MDP-Containing Adhesives Following Pre-Operative and Post-Operative Dental Bleaching: An In Vitro Study

**DOI:** 10.3390/jfb17100516

**Published:** 2026-10-15

**Authors:** Danya Hashem, Lamis A. Al-Taee, Khaled E. Ahmed, Avijit Banerjee, Somaya Ali Saleh

**Affiliations:** 1 Department of Restorative Dental Science, College of Dentistry, Taibah University, Madinah, Saudi Arabia; sosaleh@taibahu.edu.sa; 2 Department of Conservative and Aesthetic Dentistry, College of Dentistry, University of Baghdad, Baghdad, Iraq; lamis.al-taee@codental.uobaghdad.edu.iq; 3 Melbourne Dental School, University of Melbourne, Melbourne, Australia; khaled.ahmed@unimelb.edu.au; 4 Centre for Oral, Clinical & Translational Sciences, Faculty of Dentistry, Oral & Craniofacial Sciences, King’s College London, London, UK; avijit.banerjee@kcl.ac.uk; 5 Department of Operative Dentistry, Faculty of Dentistry, Ain Shams University, Cairo, Egypt

**Keywords:** 10-MDP adhesives, bleaching, carbamide peroxide, hydrogen peroxide, micro-tensile bond strength, sodium ascorbate

## Abstract

Dental bleaching may compromise resin–tooth bonding, while functional monomers and post-bleaching strategies may influence adhesive performance. This study assessed the micro-tensile bond strength (µTBS) of a 10-MDP-containing versus a non-10-MDP-containing adhesive on enamel and dentine following hydrogen peroxide (HP) or carbamide peroxide (CP) bleaching performed before bonding or after restoration aging. Forty human molars yielded 400 beams for µTBS testing. For pre-operative bleaching, specimens received 32% HP or 35% CP and were bonded immediately, after a one-week delay, or immediately after sodium ascorbate (SA) application. For post-operative bleaching, bonded specimens were aged for three months before HP or CP bleaching; non-bleached specimens served as controls. Failure modes were assessed stereomicroscopically, and data were analyzed using four-way ANOVA. The tested 10-MDP-containing adhesive showed higher µTBS across experimental groups. HP produced higher µTBS in SA-treated and post-operative groups with the 10-MDP-containing adhesive, whereas CP produced higher µTBS after a one-week delay. Immediate bonding after bleaching produced the lowest µTBS values. Within the study conditions, bond strength was influenced by adhesive system, bleaching protocol, and bonding timing; SA after HP and delayed bonding after CP were associated with more favorable outcomes.

## 1. Introduction

The growing trend for aesthetic dentistry fuelled by social media, celebrity influence, and the growing desire for a polished, camera-ready appearance has led to a surge in dental bleaching procedures [1]. Vital bleaching is considered as one of the most conservative treatment options, as it requires no removal of tooth structure and delivers rapid, effective results [2]. Different concentrations of hydrogen peroxide (H_2_O_2_; HP) and carbamide peroxide (CP) are the active substances of bleaching agents used either at home or in the office supervised by a dentist [3]. However, it does come with many undesirable side-effects, one of which is reduced bond strength of resin composite to tooth structure. This effect has been linked to residual oxygen remaining on the tooth surface, which disrupts resin bonding and inhibits complete polymerization [3]. Other studies have suggested that vital bleaching can modify the protein and mineral composition of the superficial enamel layers, potentially leading to a reduction in bond strength [4,5]. After the bleaching treatment, patients sometimes require aesthetic procedures such as laminate veneers or restorations for repairing deficiencies (pre-operative dental bleaching) [3]. In other situations, bleaching is performed even when the patient already has existing resin composite restorations (post-operative dental bleaching). Various approaches have been suggested to reduce the negative effects of bleaching on the bond strength of resin composite restorations. A commonly recommended method is to delay the bonding procedure for a period of 1 to 3 weeks after the bleaching treatment. This delay enables the elimination of residual substances due to the buffering effect of saliva. However, this method might not be practical for every patient, especially those with limited time availability [4]. An alternative approach involves applying antioxidants to neutralize residual oxygen radicals on the tooth surface, thereby re-establishing the adhesive interface [6]. Although various antioxidants have been proposed in the literature, sodium ascorbate is commonly used to reverse the adverse effects of bleaching on bond strength and has been investigated at different concentrations and with different application protocols [6]. Sodium ascorbate (SA) can neutralize free reactive radicals, and when applied immediately after bleaching procedures, it may recover the original (pre-bleaching) bond strength values or those observed 7 days after bleaching [7]. A 32% sodium ascorbate hydrogel has also been reported to improve the bond strength of resin composite to bleached enamel following short application periods [8].

However, the adhesive system type plays an important role in the outcome of enamel bond strength after the antioxidant application [8]. A recent study found that bonding agents containing acetone as a solvent showed greater bond strength values than those containing ethanol as a solvent when used on bleached enamel surfaces treated with sodium ascorbate gel [8]. Other studies compared the quality of the bond between etch-and-rinse and self-etch adhesives in both pre- and post-operative bleaching and found that total-etch adhesives provided higher bond strength values compared to self-etch adhesives [9,10]. However, no studies have investigated the effect of the functional monomer on the bond strength values of bleached enamel and dentine specimens, whether bonding was done before or after bleaching. Bonding agents that contain the functional monomer 10-MDP (10-methacryloyloxydecyl dihydrogen phosphate) are known to form a stable chemical bond with hydroxyapatite [11]. Moreover, they produce a durable adhesive interface characterized by low calcium salt dissolution and a long hydrophobic chain that creates an MDP–Ca salt layer, thereby enhancing bond strength and long-term stability [11].

Furthermore, the majority of previous studies evaluating the effect of dental bleaching on adhesive performance have focused primarily on enamel; however, the inclusion of dentine is also clinically relevant. Bleaching agents diffuse through enamel and reach the underlying dentine, where they interact with a substrate that differs substantially in composition, permeability, water content, and bonding characteristics. The collagen-rich organic matrix of dentine may be more susceptible to oxidative changes induced by bleaching agents, potentially influencing adhesive infiltration and bond durability [12,13]. Furthermore, dentine may be directly exposed to bleaching agents in clinical situations involving wear, erosion, gingival recession, non-carious cervical lesions, deep cavity preparations, or exposed root surfaces. Therefore, evaluating the effects of bleaching on both enamel and dentine provides a more comprehensive assessment of adhesive performance under clinically relevant conditions and improves the translational value of the findings.

While the effects of pre-operative dental bleaching on adhesive bonding have been extensively investigated, limited evidence is available regarding the evaluation of both pre-operative and post-operative bleaching protocols within the same experimental framework. Evaluating both scenarios within the same study allows the performance of the same adhesive systems and dental substrates to be characterized under two clinically relevant sequences of bleaching and restorative treatment. Therefore, the present study provides an assessment of the bond strength of 10-MDP-containing adhesives under two clinically relevant bleaching scenarios, contributing to a broader understanding of adhesive performance before and after restorative treatment.

The aims of the study are as follows:

1. To assess the micro-tensile bond strength (µTBS) of 10-MDP-containing adhesive compared to a non-10-MDP-containing adhesive applied on enamel and dentine substrates immediately after bleaching using hydrogen peroxide (HP) versus carbamide peroxide (CP) compared to one week after bleaching and after the application of sodium ascorbate immediately after bleaching (pre-operative dental bleaching).
2. To assess the µTBS of 10-MDP-containing adhesive compared to a non-10-MDP-containing adhesive applied on enamel and dentine substrates and aged for 3 months followed by bleaching using HP versus CP as bleaching agents after the aging period (post-operative dental bleaching).

These clinical scenarios were investigated independently and were not intended to constitute a direct comparison between pre-operative and post-operative bleaching.

The first null hypothesis states that there is no difference in the µTBS between 10-MDP- and non-10-MDP-containing adhesives applied on enamel and dentine substrates immediately after bleaching using HP versus CP compared to one week after bleaching and after the application of sodium ascorbate immediately after bleaching. The second null hypothesis states that there is no difference in the µTBS of 10-MDP- and non-10-MDP-containing adhesives applied on enamel and dentine substrates and aged for 3 months followed by bleaching using HP versus CP.

## 2. Materials and Methods

### 2.1. Ethical Approval and Sample Size Calculation

Ethical approval for this study was granted from Taibah University College of Dentistry Research Ethics Committee (TUCDREC/240925/DHashem). A Monte Carlo simulation-based power analysis was conducted to determine the biological sample size required to detect an interaction between bleaching protocol and adhesive system on bond strength. Data were simulated 2000 times using summary data from a previous study [14], under the assumed study design, and a two-way ANOVA model was applied to each simulated dataset. The minimum sample size was defined as that providing at least 80% power at α = 0.05. The resulting Cohen’s f for the interaction term was 0.709, yielding a minimum required sample size of 40 molars. The analysis was performed using the Superpower package in the R statistical software, version 4.5.1 for Windows (R Core Team, 2025 [15]). R: A language and environment for statistical computing. R Foundation for Statistical Computing, Vienna, Austria. URL https://www.R-project.org/). Accordingly, 40 molars were included in the study.

### 2.2. Sample Grouping

A total of 40 human molars, which had been extracted at most 4 months before the start of this study and stored in saline solution, were used. The teeth did not have any previous restorations, caries, and cracks. All tooth surfaces were cleaned of debris and soft tissue remnants and then immersed in a 0.1% thymol solution. Twenty-four hours prior to the experiment, the teeth were removed from the thymol, thoroughly rinsed, and stored in distilled water. The molars were divided into 3 main groups: (1) pre-operative dental bleaching: group A (*n* = 24), which included three pre-operative treatment subgroups (A1, A2, and A3; *n* = 8 each), yielding 240 beams for µTBS testing, (2) post-operative dental bleaching: group B (*n* = 8) yielding 80 beams for µTBS testing, and (3) non-bleached control: group C (*n* = 8) yielding 80 beams for µTBS testing.

Group A was subdivided into the following subgroups:

(A1) Immediate placement of the adhesive after bleaching using either HP or CP agents on enamel or dentine samples using either 10-MDP- or non-10-MDP-containing adhesives.

(A2) Delayed placement of the adhesive after 1 week from bleaching using either hydrogen peroxide or carbamide peroxide bleaching agents on enamel or dentine samples using either 10-MDP- or non-10-MDP-containing adhesive.

(A3) immediate placement of the adhesive after treating the bleached surface (with HP or CP bleaching agents) with sodium ascorbate on enamel or dentine samples using either 10-MDP- or non-10-MDP-containing adhesive.

Group B was subdivided into enamel and dentine samples which were restored with either 10-MDP- or non-10-MDP-containing adhesive and aged for 3 months, following which bleaching using HP or CP bleaching agents was conducted after the aging period.

**Figure 1:**
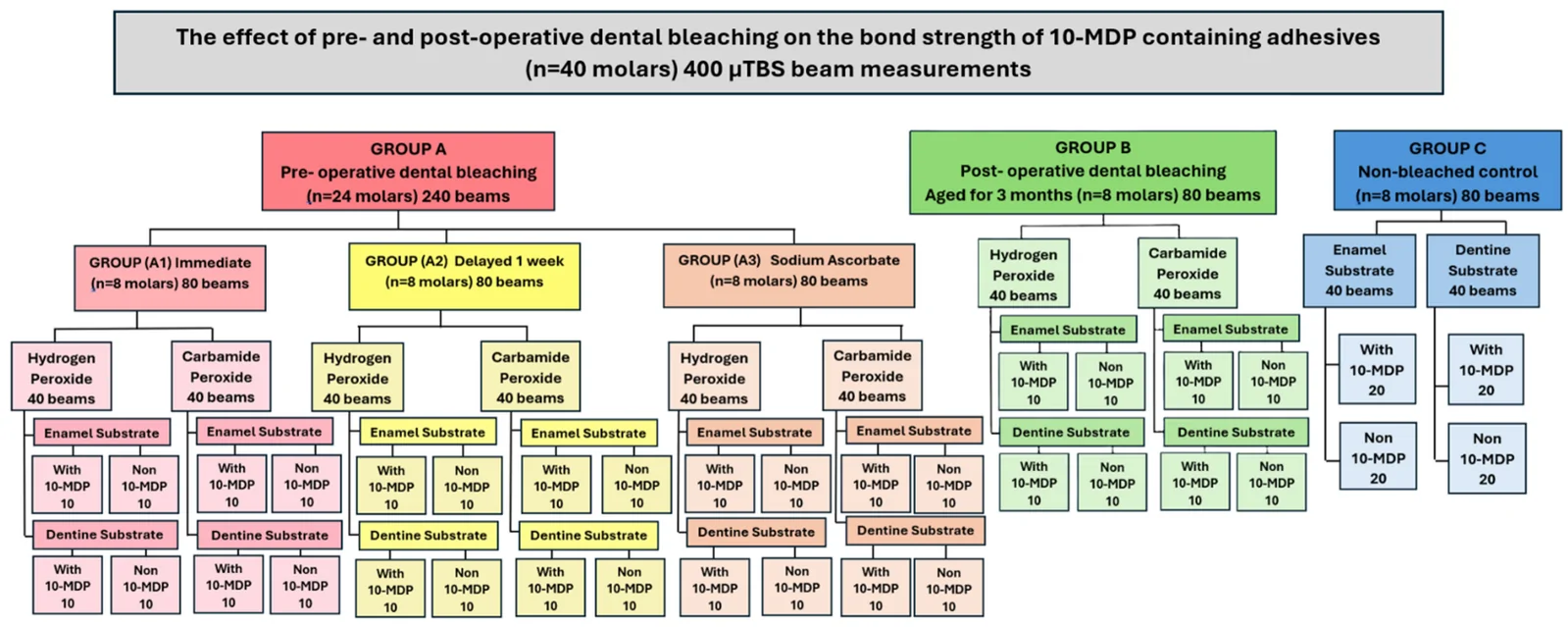
Flow chart illustrating the distribution of specimens into groups and subgroups.

Group C comprised non-bleached enamel and dentine control samples restored with either 10-MDP- or non-10-MDP-containing adhesive, with the resulting beams allocated into separate subsets for comparison with the corresponding HP and CP groups (Figure 1).

### 2.3. Sample Preparation

**Figure 2:**
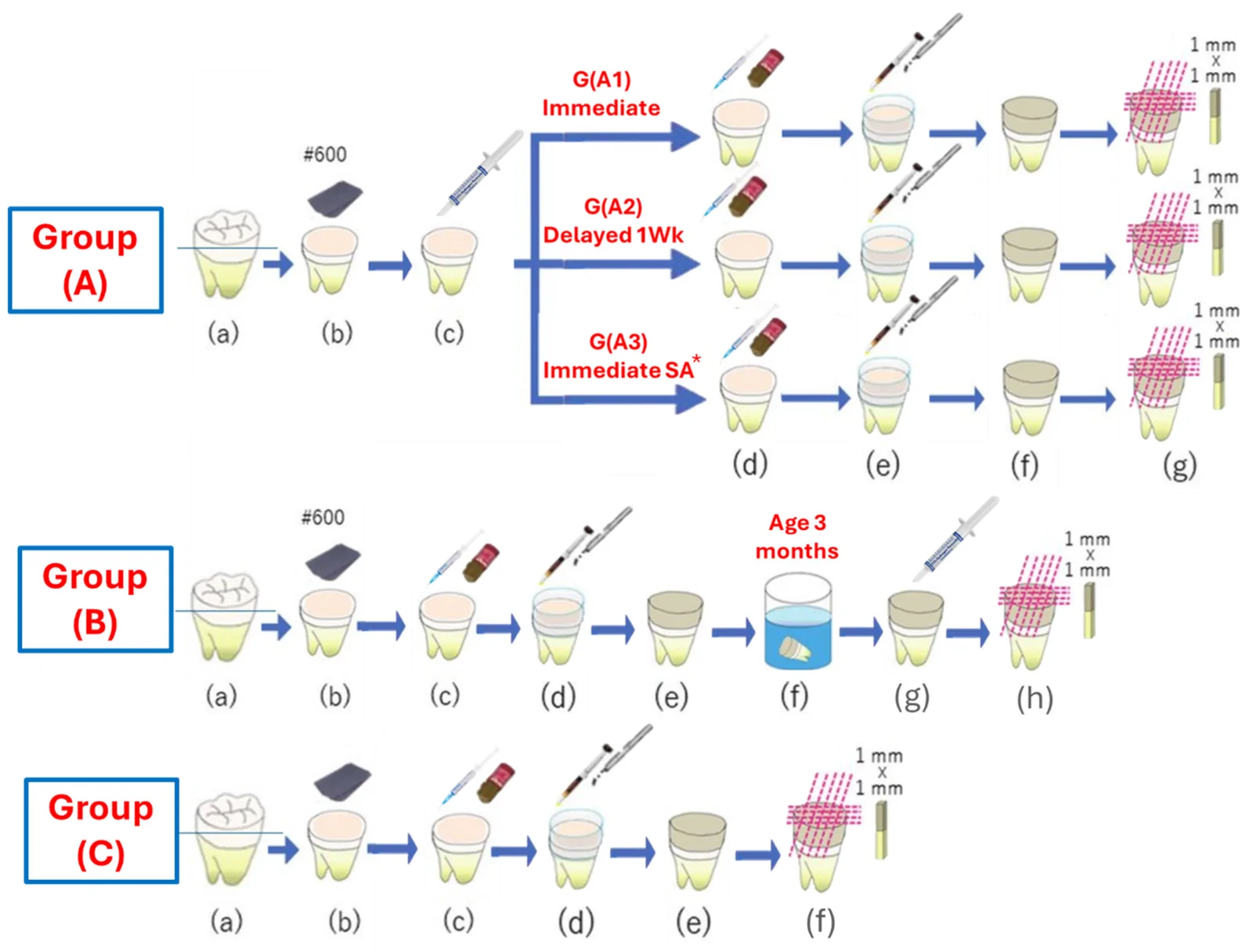
Experimental design for the dentine specimens. The same experimental design was followed for enamel specimens on the wet-ground buccal enamel surface. Group A: (a) the crown is sectioned exposing the dentine surface; (b) the sectioned half is wet-ground; (c) the flat surface of dentine is bleached using hydrogen peroxide (HP) or carbamide peroxide (CP); (d) etching and bonding of the surface according to the 3 subgroups; (e) resin composite application and polymerization; (f) samples are ready for micro-tensile preparation; (g) sectioning of the samples into beams. Group B: (a) the crown is sectioned exposing the dentine surface; (b) the sectioned half is wet-ground; (c) etching and bonding of the flat surface; (d) resin composite application and polymerization; (e) samples are ready for aging; (f) aging of the samples for 3 months: (g) bleaching of the samples using HP or CP; (h) sectioning of the samples into beams. Group C: (a) the crown is sectioned exposing the dentine surface; (b) the sectioned half is wet-ground; (c) etching and bonding of the flat surface; (d) resin composite application and polymerization; (e) samples are ready for micro-tensile preparation; (f) sectioning of the samples into beams. Figure adapted from Yamanaka et al. 2021. [16] * Sodium ascorbate.

For enamel specimens, the buccal enamel surface of the crowns were wet-ground with 180-, 220-, 400- and 600-grit silicon carbide (SiC) abrasive papers (Orientcraft, Lianyungang, China) in order to create a smooth, flat surface while preserving the enamel substrate and rinsed thoroughly using ultrasonication (Acme (Shenzhen) Technology Co. Ltd., Shenzhen, China). For dentine specimens, the crown was sectioned in half using a flexible diamond disc (NTI^®^ Superflex, NTI-Kahla GmbH, Kahla, Germany) at low speed and under water cooling, as shown in Figure 2 (a) [16]. The other occlusal half was discarded. Then, the sectioned surfaces of the crowns were wet-ground with 180-, 220-, 400- and 600-grit SiC abrasive papers (Orientcraft, Lianyungang, China) in order to create a smooth, flat surface and rinsed thoroughly using ultrasonication (Acme (Shenzhen) Technology Co. Ltd., Shenzhen, China) (Figure 2 (b)) [17]. All the materials used in the study are listed in Table 1.

For group A (*n* = 24), 12 teeth were bleached using hydrogen peroxide (Opalescence Boost 40% Ultradent, INC, South Jordan, UT, USA) and 12 teeth with carbamide peroxide (Opalescence 35% Carbamide peroxide, Ultradent, INC, South Jordan, UT, USA). The final hydrogen peroxide (HP) concentration was 32%, applied for 45 min (3 applications of 15 min) to the enamel and dentine surfaces, while the final carbamide peroxide (CP) concentration was 35%, applied for 30 min according to the manufacturer’s instructions. The concentrations and application times followed the specified protocols for the respective commercial bleaching products (Figure 2, group A (c)). After bleaching, the specimens were rinsed with deionized water. In group A1, etching was done immediately after bleaching using 37% phosphoric acid (Actino Gel Prevest, DenPro, Ltd., Jammu, India) for 30 s in enamel and 15 s in dentine and rinsed for 30 s. Excess moisture was removed using absorbent paper. Then, 10-MDP-containing adhesive (Prime & Bond Universal, Dentsply Sirona, Konstanz, Germany) and a non-10-MDP-containing adhesive (BeautiBond Universal, Shofu, Kyoto, Japan) were applied according to the manufacturer’s instructions to the surface of the tooth (Figure 2, group A (d)). After photo-activating the bonding agent for 10 s (Ivoclar Vivadent Inc., Amherst, NY, USA) (≥1400 mW/cm^2^), the surface was filled with two increments of resin composite (Beautifil Flow Plus F00, Shofu, Japan), with approximately 2 mm each employed through an incremental technique, up to approximately 4 mm high, and then photo-activation was repeated for 40 s for each layer (Figure 2, group A (e)). The output irradiance of the curing unit was calculated after every 5 specimens and confirmed with a digital power meter (Model 100 curing radiometer, Kerr, Brea, CA, USA). In group A2, the same procedure mentioned above was conducted, albeit one week after bleaching. The samples were stored in artificial saliva during the one-week period. In group A3, sodium ascorbate was applied to the enamel and dentine surfaces immediately after bleaching, followed by the same procedure detailed previously. Sodium ascorbate solution (32%) was prepared by dissolving sodium ascorbate in pure water at normal room temperature. Carbopol gel [2.5% (wt/wt)] was prepared by mixing carbopol resin with sodium ascorbate solution. The mixture was thickened by stirring and then neutralized adding triethanolamine (TEA), and the sodium ascorbate hydrogel with pH adjusted to 7.32% was applied twice for 1 min each on the enamel and dentine surfaces of the specimens. The specimens were immersed for 10 min in distilled water to dissolve sodium ascorbate on the enamel and dentine surface [8].

In group B (*n* = 8), the surfaces were etched and adhesive and resin composite applied in the same protocol described above. The specimens were stored in distilled water at 37 °C for 3 months. Then, bleaching was conducted using either 32% HP applied for 45 min (3 applications of 15 min) or 35% CP applied for 30 min to the resin composite/enamel and dentine surfaces, and these were then rinsed with deionized water (Figure 2, group B).

In the group C control (*n* = 8), the surfaces were etched, and the adhesive and resin composite were applied using the same protocol described above. These specimens served as non-bleached, non-aged controls and were stored in distilled water at 37 °C for 24 h before testing (Figure 2, group C).

### 2.4. Micro-Tensile Testing

For the micro-tensile test, each specimen was mounted in auto-polymerizing acrylic resin using a square silicone mold. The tooth was sectioned in X- and Y-axis directions by a low-speed diamond saw (Isomet 4000, Buehler Ltd., Lake Bluff, IL, USA) under air and water coolant to obtain 10 rectangular beams with a cross-sectional area of 1 mm^2^. A digital caliper (Total tools, Suzhou, China) with a precision of 0.01 mm was used to accurately measure the beam dimensions. The beams were thoroughly checked, and any defects or cracked beams were discarded. Each beam was secured to a Ciucchi’s jig using cyanoacrylate adhesive and subjected to tensile testing at a crosshead speed of 1 mm/min in a desktop testing machine until failure occurred. The fractured specimens were then carefully detached from the jig. The μTBS was calculated in megapascals (MPa) by dividing the force applied at fracture (N) by the bonded area (mm^2^).

### 2.5. Stereomicroscopy

To determine the mode of failure, all debonded surface samples were examined under a stereomicroscope (MA 100, Nikon, Melville, NY, USA) at 30× magnification. These were divided into four categories: mixed, adhesive, cohesive within resin composite, and cohesive within the substrate.

### 2.6. Scanning Electron Microscopy (SEM) Surface Evaluation

SEM was used to evaluate the surface of selected samples representing the different modes of failure. The specimens were examined without conductive coating under low-vacuum conditions using a Quanta 250 field-emission scanning electron microscope (FEG-SEM; FEI Company, Eindhoven, The Netherlands). An accelerating voltage of 20 kV was used, with magnifications ranging from 200× to 1000×. Both backscattered electron and secondary electron imaging modes were employed using the variable-contrast detector (vCD) and large-field detector (LFD), respectively. Detector selection and magnification were adjusted according to the morphological features of interest to facilitate the identification and characterization of the different failure modes.

### 2.7. Statistical Analysis

Continuous µTBS data are reported as mean ± standard deviation. Normality and homogeneity of variances were assessed using Shapiro–Wilk’s and Levene’s tests, respectively. The Shapiro–Wilk test showed no significant deviation from normality in 38 of the 40 experimental groups (*p* > 0.05), with only two groups showing marginally significant deviations (W = 0.8411, *p* = 0.045 and W = 0.8433, *p* = 0.048). Levene’s test indicated no significant heterogeneity of variances (F (39, 360) = 1.27, *p* = 0.140).

A four-way analysis of variance (ANOVA) was employed to evaluate the effects of bleaching material, substrate, adhesive system, and treatment protocol on the beam-level µTBS measurements. Treatment protocol was modelled as a categorical factor comprising five clinically relevant experimental conditions (immediate bonding, 1-week-delayed bonding, sodium ascorbate application, post-operative bleaching after aging, and non-bleached control). Post hoc comparisons of estimated marginal means were conducted using the model’s error term, with *p*-values adjusted for multiple comparisons via Holm’s method. Effect sizes were calculated and interpreted following Cohen, J. [18,19]. Statistical significance was set at α = 0.05. All analyses were performed using the R statistical software, version 4.5.1 for Windows (R Core Team, 2025) [15].

## 3. Results

The four-way ANOVA demonstrated a significant interaction among bleaching material, substrate, adhesive system, and treatment protocol (*p* < 0.001; Table 2).

### 3.1. Effect of Bleaching Material

CP achieved higher bonding strength than HP in group A1 samples bonded using MDP on both substrates, group A2 samples bonded with 10-MDP and non 10-MDP samples on both substrates, group A3 samples bonded with non-10-MDP-based adhesive to enamel, and group B samples bonded with non-10-MDP-based adhesive to dentine. HP achieved higher bonding strength than CP in group A3 samples bonded using 10-MDP-based adhesive to both substrates and group B samples bonded with 10-MDP-based adhesive to both substrates and with non-10-MDP-based adhesive to enamel only (Table 3).

### 3.2. Effect of Substrate

Enamel had significantly higher bond strength than dentine in group A2 samples that were bonded using non-10-MDP-based adhesive and bleached using CP, group A3 samples bonded with 10-MDP- and non 10-MDP-based adhesives and bleached using CP, and group B samples bonded with non-10-MDP-based adhesive and bleached using HP.

Dentine had significantly higher bond strength than enamel in group A1 samples using a non-10-MDP-based adhesive and bleached with HP, group B samples using the 10-MDP-containing adhesive and bleached with CP, and the non-bleached group C control subsets corresponding to both HP and CP comparisons using the 10-MDP-containing adhesive. Other comparisons were not statistically significant (Table 3).

### 3.3. Effect of Treatment Protocol

There was a significant difference between the bond strength measured with different treatment protocols within different variables. For samples bleached with HP and bonded to enamel using both types of adhesives, pairwise comparisons showed that group B samples had significantly higher bond strength compared to other groups. For samples bleached with HP and bonded to dentine using 10-MDP-based adhesive, pairwise comparisons showed that group B had significantly higher bond strength than other groups.

For samples bleached with CP and bonded to enamel using both types of adhesives, pairwise comparisons showed that group A2 had significantly higher bond strength than other groups. For samples bleached with CP and bonded to dentine using 10-MDP-based adhesive, pairwise comparisons showed that group A2 samples had the highest bond strength while group B bonded using the non-10-MDP-based adhesive had the highest bond strength (Table 4).

### 3.4. Effect of Adhesive Type

**Figure 3:**
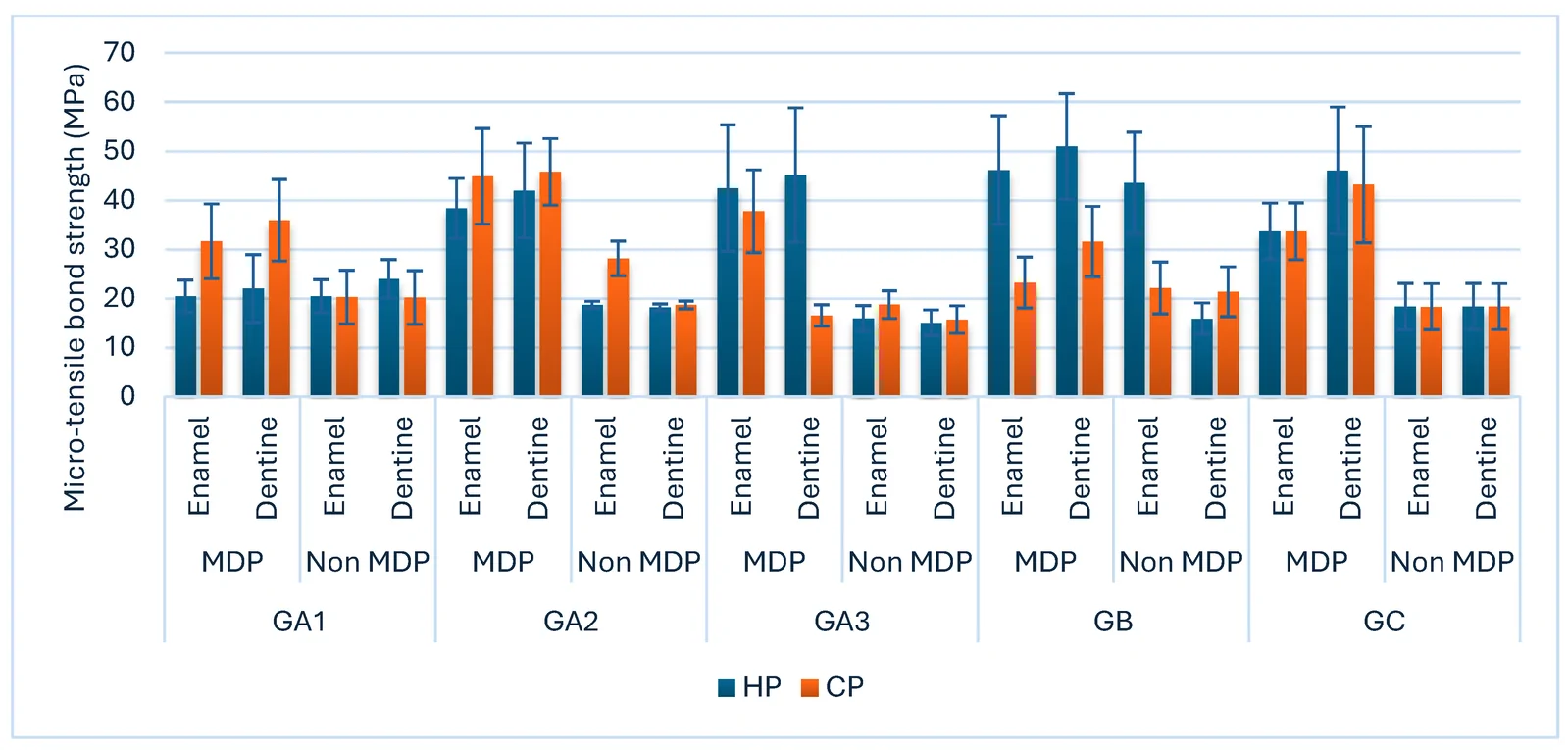
Bar showing mean and standard deviation values (error bars) for bond strength (note: the blue and orange bars in group C (GC) represent control specimens allocated for comparison with the hydrogen peroxide and carbamide peroxide groups and do not indicate bleaching treatment).

The 10-MDP-based adhesive achieved significantly higher bond strength than the non the 10-MDP-based adhesive except for in group A1 bleached with HP on dentine; however, this difference was not significant (Table 4). A summary of the bond strength values for all the groups is displayed in Figure 3.

### 3.5. Stereomicroscope Examination

**Figure 4:**
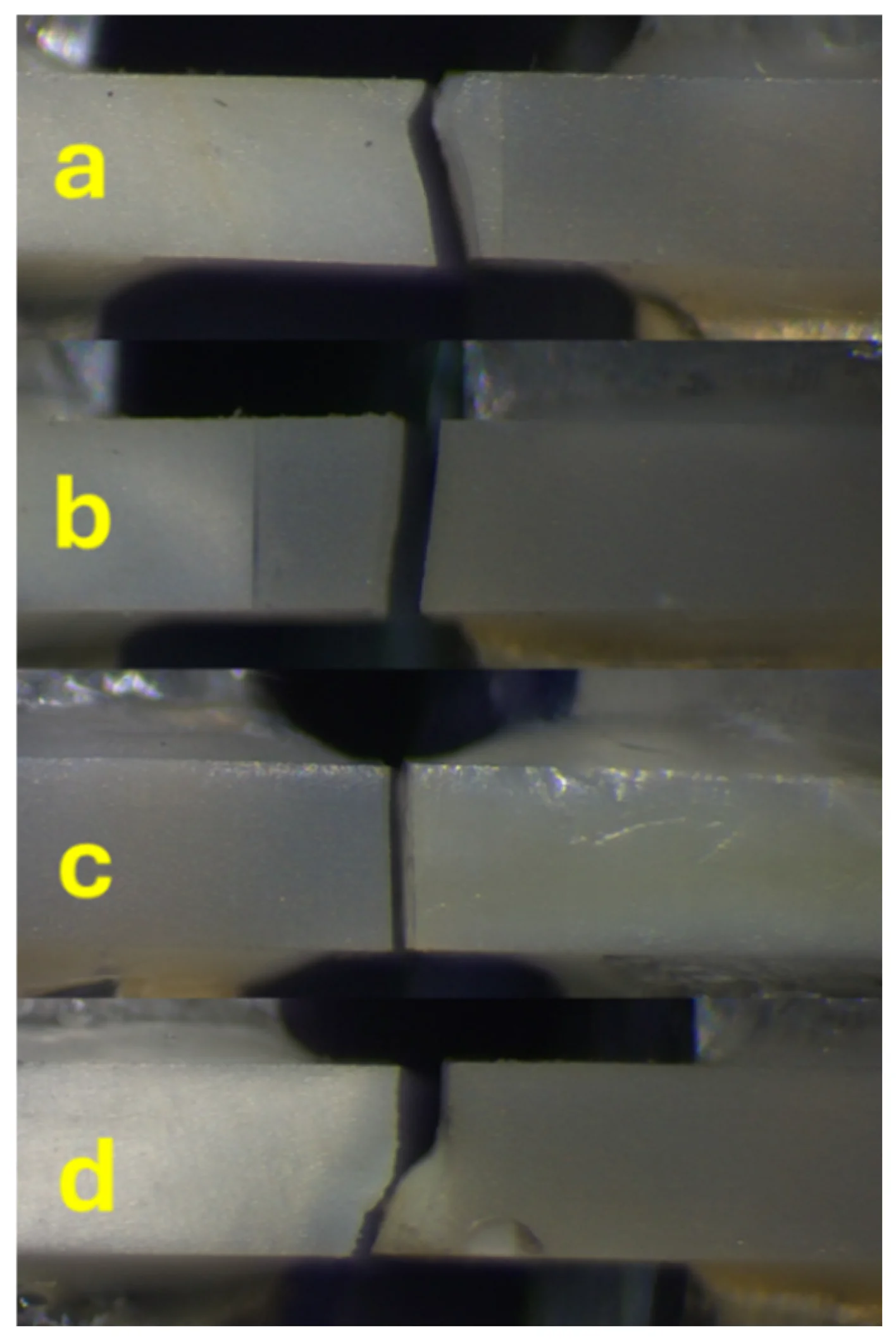
Failure modes classified into (**a**) cohesive failure within the substrate (enamel or dentine); (**b**) cohesive resin composite failure; (**c**) adhesive failure; and (**d**) mixed failure.

**Figure 5:**
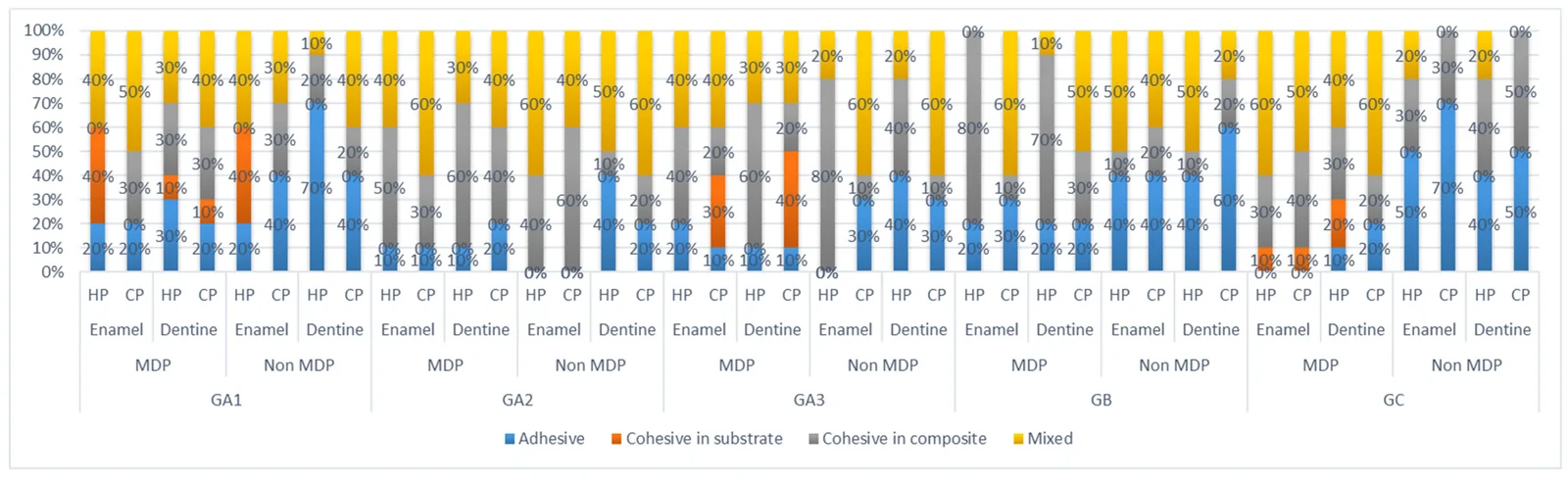
Stacked bar chart displaying failure mode distribution.

Examination using a stereomicroscope revealed four types of failure (Figure 4). The most common type of failure found in many of the groups was mixed failure, especially in samples bleached using CP, followed by cohesive failure within the resin composite. The least common type of failure was cohesive within the substrate followed by adhesive failure. The highest percentage of adhesive failure was observed in group A1 bleached with HP and bonded using non-10-MDP-based adhesives within dentine (70%) and group B bleached with CP and bonded using non-10-MDP-based adhesives within dentine (60%). The highest percentages of cohesive failure within resin composite (more than 50%) were in group A2, A3, and B, specifically in the samples bleached using HP (Figure 5).

### 3.6. SEM Analysis

**Figure 6:**
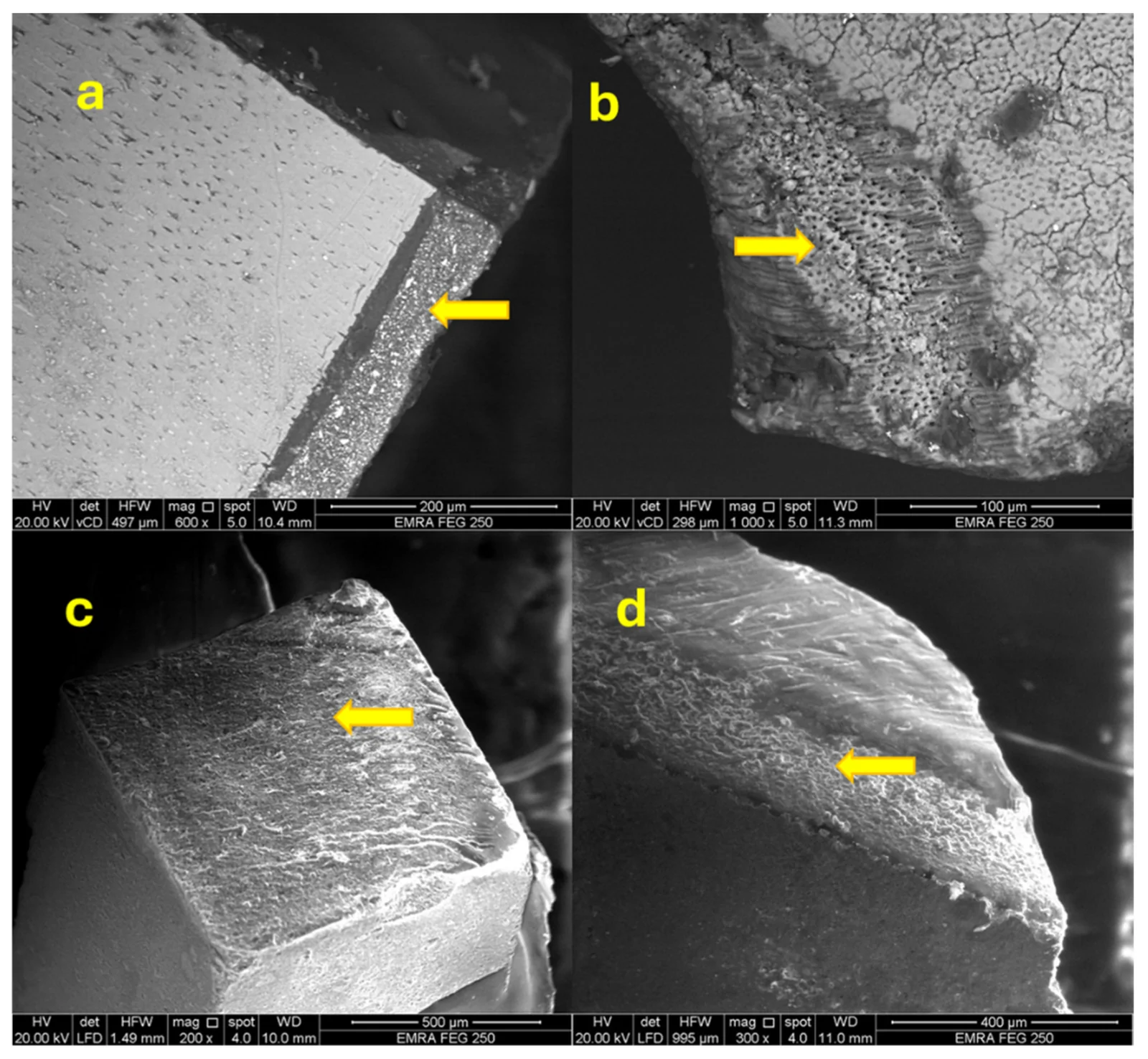
Representative scanning electron micrographs illustrating the different failure modes: (**a**) cohesive failure within resin composite (arrow: resin composite); (**b**) cohesive failure within dentine (arrow: dentinal tubules); (**c**) adhesive failure (arrow: adhesive interface); and (**d**) mixed failure (arrow: adhesive substrate). Micrographs (**a**,**b**) were acquired using the variable-contrast detector (vCD; backscattered electron imaging), whereas micrographs (**c**,**d**) were acquired using the large-field detector (LFD; secondary electron imaging).

SEM analysis of the represented samples showed cohesive failure within the resin composite (Figure 6a) characterized by the presence of rough, fibrillated fracture surfaces within the bulk of the material, indicating that failure occurred through the internal matrix rather than along the adhesive–substrate interface. Cohesive failure within dentine was also identified (Figure 6b), which was characterized by irregular crack propagation and the presence of exposed dentinal tubules. Adhesive failure can be seen (Figure 6c), with separation occurring at the interface between the adhesive and the substrate. The surface appears relatively smooth with minimal residual adhesive, indicating poor interfacial bonding. Mixed failure is apparent in Figure 6d, with fracture propagating through a combination of adhesive-underlying dentine and composite restoration. The micrograph typically shows areas where the adhesive resin remained partially attached to the dentine, alongside regions where composite remnants adhered to the adhesive layer.

## 4. Discussion

The first null hypothesis that states there is no difference in µTBS between 10-MDP- and non-10-MDP-containing adhesives applied on enamel and dentine substrates immediately after bleaching using HP versus CP compared to one week after bleaching and after the application of sodium ascorbate immediately after bleaching has been rejected. Significantly higher bond strength values were generally observed with the tested 10-MDP-containing adhesive compared with the tested non-10-MDP-containing adhesive. This effect may be due to its combined capability for chemical and micromechanical adhesion, where the functional monomer 10-MDP chemically interacts with the hydroxyapatite at the adhesive interface to form stable MDP–Ca salts. This enhances both the mechanical integrity and hydrolytic stability of the hybrid layer [20]. Additionally, the self-assembled nanolayered structures formed by 10-MDP monomers in both enamel and dentine are believed to contribute significantly to adhesive stability. These structures create a more water-resistant interface that enhances adhesion, which, together with the formation of stable MDP–Ca salts, may have contributed to the favorable bond strength observed with the tested 10-MDP-containing adhesive [11]. However, it is important to note that the two adhesive systems evaluated in this study differed not only in the presence of 10-MDP but also in other compositional characteristics, including functional monomers, solvent systems, and HEMA content. Therefore, the differences in µTBS observed between the two adhesive systems should be interpreted as reflecting their overall formulations and cannot be attributed exclusively to the presence of 10-MDP.

In this study, the lowest bond strength values within the 10-MDP-based adhesive samples were the ones which were bonded immediately after bleaching. Hydrogen peroxide and carbamide peroxide produce oxygen-rich free radicals, which diffuse into enamel and dentine and interfere with the free-radical polymerization reaction of resin adhesives and composites when applied immediately after bleaching [4]. This results in incomplete conversion of monomers, weaker resin, and resin–enamel/dentine bonding. Other studies have reported that alterations in the morphological, compositional, and mechanical properties of bleached enamel may play a role in the observed reduction in bond strength [21,22]. The increased porosity, superficial loss of mineral content, and reduction in the density and length of resin tags collectively impair micromechanical interlocking, affecting the bond strength [22]. A study investigated the effect of sodium ascorbate and delayed bonding on the bond strength of a non-10-MDP-based silorane adhesive and a two-step 10-MDP-containing self-etch adhesive system in enamel bleached with 35% HP. The 10-MDP-based adhesive system had significantly higher bond strength values compared to the silorane non-10-MDP-based adhesive, which is consistent with the results of our study [23]. Furthermore, the lowest bond strength values were found when adhesives were applied immediately after bleaching [23].

When comparing the effect of the bleaching material, HP and CP samples yielded mixed results. Samples bleached with HP had higher bond strength values when treated with sodium ascorbate and restored immediately after bleaching, while samples bleached with carbamide peroxide had higher bond strength values when restored one week after bleaching. This may be explained by the differences in peroxide breakdown and oxidant release between HP and CP. Chemically, carbamide peroxide is a complex of hydrogen peroxide and urea. It decomposes more slowly than straight hydrogen peroxide, releasing HP over a longer period of time. This slower release means CP may produce residual oxidants that persist longer in enamel/dentine compared to directly applied HP. That prolonged presence of reactive oxygen may require a longer wait to dissipate or a stronger/more prolonged antioxidant application in some conditions. HP, on the other hand, breaks down quickly and produces a large bolus of reactive oxygen that may be more readily neutralized if sodium ascorbate is applied shortly after bleaching [23,24,25].

In a study by Ismail et. al, higher µTBS values were observed for samples bleached with 35% HP and treated with sodium ascorbate and restored immediately with resin composite compared to bleached samples which were restored immediately without sodium ascorbate [12]. Another study found no difference in µTBS between samples bleached with 6% HP and 10% and 16% CP followed by treatment with sodium ascorbate and then restoration with resin composite [26]. This may be attributed to the low concentrations of the bleaching agents used. On the other hand, another study compared the µTBS of samples bleached with 35% HP versus 10% CP and treated with sodium ascorbate and restored immediately with resin composite. The results demonstrated that samples bleached with CP had higher µTBS than those bleached using HP [27]. This can be explained by the lower percentage of CP compared to the percentage of CP used in this research study where it is reported that 10% carbamide peroxide releases 3% hydrogen peroxide and 7% urea, which is 10 times lower than the 35% concentration of carbamide peroxide [8].

Furthermore, higher bond strength values were observed in the samples which were bleached post-operatively using HP compared to CB, thus rejecting the second null hypothesis of this study. The post-operative bleaching protocol was designed to represent a clinically relevant treatment scenario in which existing restorations are exposed to bleaching agents after a period of service. Therefore, the observed outcomes reflect the response of aged restorations to subsequent bleaching and should be interpreted within this clinical context.

Water aging is known to promote hydrolytic degradation of the resin–dentine interface through water sorption, plasticization of the resin matrix, and degradation of exposed collagen fibrils, potentially reducing bond strength over time [28]. The favorable bond strength values observed following post-operative bleaching, particularly with 10-MDP-containing adhesives, suggest that these adhesive interfaces maintained sufficient stability despite the combined challenges of aging and bleaching. This may partly be related to the sequence of treatment in the post-operative group, in which the adhesive interface was established before exposure to the bleaching agents. In pre-operative bleaching, residual oxidizing species and bleaching-induced alterations in the dental substrate may interfere with subsequent adhesive polymerization and bonding. In contrast, post-operative bleaching exposes an already-polymerized adhesive interface to the bleaching agent.

The stability of the adhesive bond between resin composite and tooth tissue is one of the main factors determining restoration longevity, which may be challenged by different factors, one of which is the chemical agents found in bleaching gels. The chemical interaction between 10-MDP and hydroxyapatite may have contributed to the favorable bond strength observed with the tested 10-MDP-containing adhesive. However, because the evaluated adhesives also differed in other compositional characteristics, the observed performance cannot be attributed exclusively to 10-MDP. Moreover, the hydrophobic resin in this two-step system may seal the primed tissues, creating a barrier that prevents water and active components from the bleaching gel from penetrating the adhesive interface [10]. Previous studies investigated the effect of different concentrations of CP on the bond strength of the restoration–dental structure and found that increasing the concentration had a detrimental effect on the bond strength [10,29]; however, there are no previous studies comparing the effect of HP and CP on the bond strength post-operatively.

When comparing the effect of the treatment protocol, immediate bonding after bleaching with no use of ascorbic acid and no waiting time produced the lowest µTBS when used with 10-MDP-based adhesives, and this is consistent with many studies which found the lowest bond strength values for the groups bonded immediately after bleaching [3,26,30,31]. Moreover, sodium ascorbate application and delaying bonding for one week were associated with more favorable µTBS outcomes than immediate bonding without antioxidant treatment. In the separate post-operative model, favorable bond strength values were observed after three months of aging followed by bleaching, particularly with the HP protocol.

The present investigation employed a micro-tensile set-up, which is a widely used method for evaluating the bond strength of dental materials and has a greater discriminative capability compared to other tests in addition to a more uniform loading stress distribution over a smaller bonded area [32]. Furthermore, a total-etch adhesive method was employed in this study rather than a self-etch adhesive method, whereas total-etch adhesives have already been proven in the literature to have higher bond strength values compared to self-etch adhesives on bleached enamel and dentine surfaces [9,10,33]. This reduced durability observed with self-etch adhesive systems is attributable not only to the presence of partially infiltrated collagen fibrils but also to their pronounced hydrophilic nature. This hydrophilicity facilitates water sorption within the adhesive interface, leading to water tree formation, polymer swelling, and plasticization of the adhesive resin matrix. This may act as stress concentrators, accelerating water penetration and diminishing adhesive joint mechanical strength [10]. Furthermore, the bond strength achieved by adhesive systems is influenced by their effectiveness in removing the smear layer and demineralizing the underlying tooth substrate following tooth preparation. In etch-and-rinse adhesive systems, high bond strength and enhanced resistance to the adverse effects of bleaching and water exposure are attributed to optimal phosphoric acid etching of enamel and dentine, which creates a highly retentive micro-porous surface conducive to effective resin infiltration and micromechanical interlocking [10]. Although phosphoric acid etching removes a substantial portion of the superficial hydroxyapatite, residual hydroxyapatite crystals surrounding collagen fibrils may remain available for interaction with functional monomers such as 10-MDP. Therefore, even when used in etch-and-rinse strategies, the presence of residual hydroxyapatite together with enhanced resin infiltration and interface stability may explain the favorable performance of 10-MDP-containing adhesives observed in the present study [11]. Interestingly, a study by Türkmen et al. found no significant difference between self-etch and total-etch adhesives used on bleached enamel, although it is imperative to point out that a 10-MDP-containing self-etch adhesive was used compared to a non-10-MDP-containing total-etch adhesive [30].

In the present study, enamel and dentine samples were included, although most studies investigating bleaching have used enamel samples only. Dentine samples were added in this study because dentine differs fundamentally from enamel in composition, permeability, and bonding mechanism. When the bleaching agent is applied to the enamel, it moves through the enamel pores and penetrates the dentine, where it interacts more readily with the collagen-rich organic matrix. This is where the majority of the chemical reactions of the bleaching process occur. Dentine can also serve as a reservoir, trapping oxygen released as a by-product of the bleaching reaction. In addition, dentine may be directly exposed to bleaching agents in clinical situations involving attrition, erosion, caries, or other defects, making the evaluation of bleaching effects on dentine clinically relevant [12,13]. In this study, enamel and dentine influenced bond strength outcomes, but this effect varies depending on other factors such as the type of adhesive, bleaching material used, and timing of restoration (immediate or delayed). These complex interactions highlight the need to consider substrate variability to optimize bond strength and long-term restoration success.

The failure modes observed in this study provide insight into the nature of the bond quality under different treatment protocols. Overall, a higher proportion of cohesive failure within resin composite and mixed failures in groups treated with antioxidants or delayed bonding suggests improved bond stability, whereas prevalent adhesive failures in groups with immediate bonding after bleaching and those with non-10-MDP-based adhesives point to weaker interfacial adhesion. These failure patterns were generally consistent with the observed µTBS findings across the different treatment conditions.

Although this study provides valuable insights, it is not without limitations. The long-term durability of the bond over extended periods or under clinically relevant aging conditions like thermocycling or mechanical loading was not investigated. The post-operative bleaching protocol included a three-month aging period before bleaching to reflect clinical practice, whereas the non-bleached control was evaluated after 24 h. Consequently, the independent effects of aging and bleaching cannot be distinguished, and the findings associated with post-operative bleaching should be interpreted within the context of aged restorations exposed to subsequent bleaching rather than as the isolated effect of bleaching alone. Multiple µTBS beams were obtained from individual molars, and measurements originating from the same tooth may therefore exhibit some degree of correlation; this should be considered when interpreting the statistical findings. In addition, the study evaluated one commercially available 10-MDP-containing adhesive and one non-10-MDP-containing adhesive. As these materials differ in their overall composition, the findings should be considered specific to the adhesive systems evaluated rather than attributed exclusively to the presence or absence of 10-MDP. Also, the HP and CP bleaching regimens evaluated in this study represented the specified application protocols of the respective commercial products and therefore differed in peroxide chemistry, effective hydrogen peroxide exposure, release kinetics, concentration, and application time. Accordingly, comparisons between HP and CP should be interpreted within the context of the specific bleaching protocols evaluated. Other concentrations, application time, and number of bleaching sessions should be explored. Furthermore, because this is an in vitro study, it cannot completely replicate the complex conditions of the oral environment, such as saliva, temperature changes, masticatory forces, pH variations, and biofilm interactions, which may influence bond durability and success in vivo. By acknowledging these limitations, further research can be directed toward addressing these gaps, including long-term clinical studies and testing under more varied and realistic conditions.

## 5. Conclusions

Within the limitations of this in vitro study, adhesive performance following dental bleaching was influenced by the adhesive system, bleaching protocol, and timing of bonding. The tested 10-MDP-containing adhesive generally demonstrated higher µTBS than the tested non-10-MDP-containing adhesive across the evaluated conditions, reflecting differences in the overall adhesive formulations. For pre-operative bleaching, the findings suggest that the bonding strategy should be considered in relation to the bleaching protocol, with sodium ascorbate following HP and a one-week delay following CP associated with more favorable bonding outcomes than immediate bonding without intervention. For post-operative bleaching of restorations aged for three months, the HP protocol was associated with more favorable µTBS outcomes than CP, particularly with the tested 10-MDP-containing adhesive, suggesting that the response of established adhesive interfaces to subsequent bleaching may vary according to the bleaching protocol. Further studies incorporating longer-term aging and clinically relevant conditions are needed to determine the durability and clinical significance of these findings.

## Figures and Tables

**Table 1:** List of materials used in the study.

Material Name and Manufacturer	Composition	Lot Number
Etchant: Actino Gel Prevest DenPro, Ltd., Jammu, India	37% Phosphoric acid, H_2_O xanthan gel.	PK2122820
10-MDP Bonding agent: Prime & Bond Universal, Dentsply Sirona, Germany	PENTA, and 10-MDP, Bis-GMA and HEMA, Water-Isopropanol solvent.	2404000914
Non-10-MDP Bonding agent: BeautiBond Universal, Shofu, Japan	Bis-GMA, TEGDMA, phosphonic acid monomer, carboxylic acid monomer, acetone, water, photo-initiators, accelerator; filler-containing, HEMA-free formulation.	112251
Resin Composite: Beautifil Flow Plus F00, Shofu, Japan	Bis-GMA, TEGDMA. S-PRG filler based on fluoro-boro-aluminosilicate-glass.	082360
Bleaching agent 1: Opalescence Boost 40% hydrogen peroxide, Ultradent, INC, USA	One barrel contains 1.1% sodium fluoride and 3% potassium nitrate. This is mixed with another barrel that contains hydrogen peroxide, resulting in a final mixture with a hydrogen peroxide concentration of 32%. pH 7.0	BW3Z1
Bleaching agent 2: Opalescence 35% carbamide peroxide, Ultradent, INC, USA	Urea NH_3_ 6.5%, which buffers the pH of H_2_O_2_ and holds it steadier, decreases its degradation and prolongs its action. Potassium nitrate and sodium fluoride, water. pH 6.5	BZMFH

PENTA: dipentaerythritol pentacrylate phosphate; 10-MDP: 10-methacryloyloxydecyl dihydrogen phosphate; HEMA: 2-hydroxyethyl methacrylate. Bis-GMA: bisphenol A-glycidyl methacrylate; TEGDMA: triethylene glycol dimethacrylate; S-PRG: surface pre-reacted glass-ionomer.

**Table 2:** Four-way ANOVA for micro-tensile bond strength (µTBS).

Source	df1	df2	F-Value	*p*-Value	ω^2^ (95% CI)
Bleaching material	1	360	18.33	<0.001 *	0.05 (0.01 to 0.09)
Substrate	1	360	0.11	0.736 ns	0.00 (0.00 to 0.00)
Adhesive	1	360	0.00	0.996 ns	0.00 (0.00 to 0.00)
Treatment protocol	4	360	18.04	<0.001 *	0.16 (0.09 to 0.22)
Bleaching material * Substrate	1	360	0.33	0.564 ns	0.00 (0.00 to 0.00)
Bleaching material * Adhesive	1	360	10.87	0.001 *	0.03 (0.00 to 0.07)
Substrate * Adhesive	1	360	0.91	0.342 ns	0.00 (0.00 to 0.00)
Bleaching material * Treatment	4	360	15.18	<0.001 *	0.13 (0.07 to 0.19)
Substrate * Treatment	4	360	0.75	0.557 ns	0.00 (0.00 to 0.00)
Adhesive * Treatment	4	360	17.86	<0.001 *	0.16 (0.09 to 0.22)
Bleaching material * Substrate * Adhesive	1	360	1.63	0.203 ns	0.00 (0.00 to 0.02)
Bleaching material * Substrate * Treatment	4	360	9.29	<0.001 *	0.08 (0.03 to 0.13)
Bleaching material * Adhesive * Treatment	4	360	4.66	0.001 *	0.04 (0.00 to 0.08)
Substrate * Adhesive * Treatment	4	360	11.33	<0.001 *	0.10 (0.04 to 0.16)
Bleaching material * Substrate * Adhesive * Treatment	4	360	7.22	<0.001 *	0.06 (0.02 to 0.11)

df: degree of freedom; CI: confidence interval; ω^2^: omega squared; * significant (*p* < 0.05); ns: not significant.

**Table 3:** Effect of bleaching material and substrate on bond strength.

Treatment Protocol	Adhesive	Substrate	Micro-Tensile Bond Strength (Mean ± SD) (MPa)		*p*-Value
			HP	CP	
Group A1: Pre-op immediate bonding	10-MDP	Enamel	20.41 ± 3.28	31.60 ± 7.61	<0.001 *
		Dentine	21.99 ± 6.89	35.90 ± 8.30	<0.001 *
		*p*-value	0.793	0.292	
	Non 10-MDP	Enamel	20.42 ± 3.37	20.25 ± 5.45	0.744
		Dentine	23.93 ± 3.97	20.16 ± 5.45	0.065
		*p*-value	0.037 *	0.971	
Group A2: Pre-op bonding after 1 week	10-MDP	Enamel	38.30 ± 6.11	44.84 ± 9.72	0.234
		Dentine	41.94 ± 9.65	45.73 ± 6.78	0.282
		*p*-value	0.462	0.778	
	Non 10-MDP	Enamel	18.66 ± 0.73	28.12 ± 3.53	<0.001 *
		Dentine	18.12 ± 0.71	18.64 ± 0.78	0.149
		*p*-value	0.106	<0.001 *	
Group A3: Pre-op immediate bonding and sodium ascorbate	10-MDP	Enamel	42.43 ± 12.89	37.73 ± 8.43	0.504
		Dentine	45.13 ± 13.68	16.50 ± 2.17	<0.001 *
		*p*-value	0.689	<0.001 *	
	Non 10-MDP	Enamel	15.87 ± 2.63	18.72 ± 2.82	0.019 *
		Dentine	15.02 ± 2.61	15.68 ± 2.81	0.619
		*p*-value	0.458	0.018 *	
Group B: Post-op bleaching after 3 months	10-MDP	Enamel	46.13 ± 11.04	23.20 ± 5.20	<0.001 *
		Dentine	50.94 ± 10.72	31.54 ± 7.18	<0.001 *
		*p*-value	0.372	0.011 *	
	Non 10-MDP	Enamel	43.49 ± 10.31	22.12 ± 5.26	<0.001 *
		Dentine	15.85 ± 3.21	21.34 ± 5.07	0.003 *
		*p*-value	<0.001 *	0.715	
Group C: No bleaching	10-MDP	Enamel	33.63 ± 5.74	33.63 ± 5.78	0.999
		Dentine	46.04 ± 12.91	43.15 ± 11.82	0.901
		*p*-value	0.023 *	0.024 *	
	Non 10-MDP	Enamel	18.31 ± 4.72	18.28 ± 4.69	0.989
		Dentine	18.30 ± 4.72	18.31 ± 4.69	0.995
		*p*-value	0.996	0.987	

SD: standard deviation. For group C, the HP and CP columns represent separate non-bleached control subsets allocated for comparison with the corresponding HP and CP groups; values with different superscripts within the same horizontal row are significantly different; * significant (*p* < 0.05).

**Table 4:** Effect of treatment protocol and adhesive on bond strength.

Material	Substrate	Adhesive	Micro-Tensile Bond Strength (Mean ± SD) (MPa)					*p*-Value
			Group A1: Pre-Op Immediate Bonding	Group A2: Pre-Op Bonding After 1 Week	Group A3: Pre-Op Immediate Bonding and Sodium Ascorbate	Group B: Post-Op Bleaching After 3 Months	Group C: No Bleaching	
*HP*	Enamel	10-MDP	20.41 ± 3.28 ^A^	38.30 ± 6.11 ^B^	42.43 ± 12.89 ^B^	46.13 ± 11.04 ^B^	33.63 ± 5.74 ^B^	<0.001 *
		Non 10-MDP	20.42 ± 3.37 ^B^	18.66 ± 0.73 ^B^	15.87 ± 2.63 ^A^	43.49 ± 10.31 ^C^	18.31 ± 4.72 ^AB^	<0.001 *
		*p*-value	0.995	<0.001 *	<0.001 *	0.709	<0.001 *	
	Dentine	10-MDP	21.99 ± 6.89 ^A^	41.94 ± 9.65 ^B^	45.13 ± 13.68 ^B^	50.94 ± 10.72 ^B^	46.04 ± 12.91 ^B^	<0.001 *
		Non 10-MDP	23.93 ± 3.97 ^C^	18.12 ± 0.71 ^B^	15.02 ± 2.61 ^A^	15.85 ± 3.21 ^AB^	18.30 ± 4.72 ^AB^	<0.001 *
		*p*-value	0.289	<0.001 *	<0.001 *	<0.001 *	<0.001 *	
*CP*	Enamel	10-MDP	31.60 ± 7.61 ^B^	44.84 ± 9.72 ^B^	37.73 ± 8.43 ^B^	23.20 ± 5.20 ^A^	33.63 ± 5.78 ^B^	<0.001 *
		Non 10-MDP	20.25 ± 5.45 ^A^	28.12 ± 3.53 ^B^	18.72 ± 2.82 ^A^	22.12 ± 5.26 ^A^	18.28 ± 4.69 ^A^	<0.001 *
		*p*-value	<0.001 *	<0.001 *	<0.001 *	0.664	<0.001 *	
	Dentine	10-MDP	35.90 ± 8.30 ^BC^	45.73 ± 6.78 ^C^	16.50 ± 2.17 ^A^	31.54 ± 7.18 ^B^	43.15 ± 11.82 ^BC^	<0.001 *
		Non 10-MDP	20.16 ± 5.45 ^AB^	18.64 ± 0.78 ^B^	15.68 ± 2.81 ^A^	21.34 ± 5.07 ^B^	18.31 ± 4.69 ^AB^	0.016 *
		*p*-value	<0.001 *	<0.001 *	0.448	<0.001 *	<0.001 *	

SD: standard deviation. For group C, the values shown within the HP and CP sections represent separate non-bleached control subsets allocated for comparison with the corresponding HP and CP groups, respectively; values with different superscripts within the same horizontal row are significantly different; * significant (*p* < 0.05).

## Data Availability

The datasets used and/or analyzed during the current study are available from the corresponding author on reasonable request.

## Abbreviations

The following abbreviations are used in this manuscript:

10-MDP	10-Methacryloyloxydecyl dihydrogen phosphate
ANOVA	Analysis of variance
Bis-GMA	Bisphenol A-glycidyl methacrylate
TEGDMA	Triethylene glycol dimethacrylate
CI	Confidence interval
CP	Carbamide peroxide
df	Degrees of freedom
EDX	Energy-dispersive X-ray analysis
FEG	Field-emission gun
HEMA	2-Hydroxyethyl methacrylate
HP	Hydrogen peroxide
10-MDP–Ca	10-MDP–calcium salt
MPa	Megapascal
PENTA	Dipentaerythritol pentacrylate phosphate
SA	Sodium ascorbate
SD	Standard deviation
SEM	Scanning electron microscopy
µTBS	Micro-tensile bond strength
S-PRG	Surface pre-reacted glass-ionomer
TEA	Triethanolamine
SiC	Silicon carbide
